# Assessing Symptom Accommodation of Social Anxiety Symptoms Among Chinese Adults: Factor Structure and Psychometric Properties of Family Accommodation Scale Anxiety—Adult Report

**DOI:** 10.3389/fpsyg.2020.01018

**Published:** 2020-06-04

**Authors:** Congmei Lou, Xiaolu Zhou, Eli R. Lebowitz, Laurel L. Williams, Eric A. Storch

**Affiliations:** ^1^Research Institute for International and Comparative Education, Shanghai Normal University, Shanghai, China; ^2^Child Study Center, Yale School of Medicine, New Haven, CT, United States; ^3^Menninger Department of Psychiatry and Behavioral Sciences, Baylor College of Medicine, Houston, TX, United States

**Keywords:** assessment, Chinese adults, factor structure, psychometric properties, reliability, social anxiety symptoms, symptom accommodation

## Abstract

**Objective:**

Symptom accommodation is an important interpersonal construct associated with more severe symptoms, lower levels of functioning, and worse treatment outcomes across various mental health conditions, including social anxiety. Research on this phenomenon is surprisingly absent in Chinese culture, where interpersonal relationships are highly emphasized. This may be due to the absence of a valid Chinese symptom accommodation measure for individuals with social anxiety symptoms. The current study aimed to examine the factor structure and psychometric properties of the Family Accommodation Scale Anxiety—Adult Report (FASA-AR) in Chinese adults.

**Methods:**

Three hundred and seventy-five Chinese undergraduate students with social anxiety symptoms completed a battery of self-report measures assessing symptom accommodation in relation to social anxiety symptoms and related impairments, as well as overall symptoms of anxiety and depression.

**Results:**

Confirmatory factor analysis supported a two-factor model of symptom accommodation, with factors named Participation in symptom-related behaviors and Modification of functioning. The multiple indicators multiple causes model indicated the indicators of the FASA-AR, mainly the participation in symptom-related behaviors subscale, were not invariant across gender. Internal consistency for the FASA-AR total score and subscale scores was good. Convergent validity of the FASA-AR was evidenced by significant positive association with ratings of social anxiety symptoms, social anxiety related impairments, and anxiety symptoms. Divergent validity was supported by non-significant relation with depression symptoms. Nearly all participants (94.7%) endorsed being accommodated to some extent in the past month.

**Conclusion:**

Symptom accommodation is an important construct and is related to social anxiety symptoms among Chinese adults. The FASA-AR demonstrated a clear two-factor latent structure and possessed good psychometric properties that can validly and reliably assess symptom accommodation of social anxiety among Chinese adults.

## Introduction

Social anxiety disorder (SAD) is a common mental health concern characterized by a marked and intense fear of social interaction and performance situations due to concern about negative evaluation, embarrassment, and/or rejection (American Psychiatric Association, 2013). Approximately 0.7% of Chinese adults are estimated to have SAD (i.e., more than 6 million people; Huang et al., 2019). Individuals with SAD experience significant interference across various aspects of their lives, including work, family, and social functioning (Schneier et al., 1994; Fehm et al., 2008).

There is an expanding body of research into the interplay between mental health problems and interpersonal dynamics. Importantly, symptom accommodation is a key element for the development, maintenance, and treatment of various anxiety disorders, including SAD (Kagan et al., 2017). Symptom accommodation is a phenomenon whereby individuals change their own behavior to help their relatives or significant others (including partners and friends) to reduce or avoid distress caused by psychiatric problems (Lebowitz et al., 2016). For individuals with social anxiety, the most frequently reported accommodation behaviors are providing reassurance, facilitating avoidance, and engaging in avoidance behaviors with the sufferers (Joogoolsingh et al., 2015). These well-intentioned behaviors are aimed at reducing the distress of sufferers. However, receiving accommodation has been associated with more severe social anxiety symptoms, as well as more impaired daily functioning (Joogoolsingh et al., 2015). Further, symptom accommodation mediated the relationship between SAD symptom severity and functional impairment (Joogoolsingh et al., 2015).

Conceptually, symptom accommodation of social anxiety symptoms could be understood under a cognitive-behavioral framework (Hofmann, 2007; Kagan et al., 2017). According to Mowrer (1960) two-stage model of the acquisition and maintenance of anxiety or fear, in the first stage, a neutral stimulus (certain social situation in terms of social anxiety) obtains fear-evoking properties through classical conditioning. In the second stage, symptom accommodation is negatively reinforced through operant conditioning. Although accommodation does help a sufferer temporarily reduce distress, or avoid anxiety-eliciting situations, it prevents habituating to the anxiety, as well as learning that the feared outcome would not happen or would be tolerable if it did. Accommodation thus inadvertently negatively reinforces the anxiety and avoidance of related social situations (Kagan et al., 2017), and conflicts with cognitive behavioral therapy of SAD (Cuijpers et al., 2016; Scaini et al., 2016).

Despite these negative effects, research on symptom accommodation of SAD has been absent in Chinese culture, where the ideas of “facilitating interpersonal relationship[s],” “concern about others,” “putting others’ needs first,” and “maintaining harmony with others” are perpetuated. These cultural values are widely spread by academic literature, social media, or descriptions of characters in classic or popular novels (Cheung et al., 1996), and empirically evidenced as an essential part of the indigenous personality (Cheung et al., 2011). It is predictable that symptom accommodation would be taken for granted by a Chinese accommodator, and explanations about the association between accommodating and worsening SAD severity would be unexpected. Because of the cultural values relevant to the construct, studies on symptom accommodation and social anxiety in Chinese adults deserve more attention.

Estimation of the effect of symptom accommodation on the maintenance and treatment of SAD requires knowledge about the conceptualization and assessment of the variable. Symptom accommodation was first systematically investigated in relation to obsessive–compulsive disorder (Calvocoressi et al., 1995) and then extended to anxiety disorders (Lebowitz et al., 2013) and other forms of psychopathology (Caporino et al., 2012; Drury et al., 2014; Fredman et al., 2014; Storch et al., 2015, 2017). There are two common groups of accommodating behaviors categories: participation in symptom-related behaviors (PAR) and modification of daily functioning (MOD; Calvocoressi et al., 1995). Indeed, this two-factor accommodation (including the correlated PAR and MOD factors) is consistently identified and validated across psychopathology (Albert et al., 2010; Storch et al., 2017), ages (Lebowitz et al., 2013, 2019), and cultures (Gomes et al., 2015; Mahapatra et al., 2017).

Measures of symptom accommodation are derived primarily from Calvocoressi et al. (1995) Family Accommodation Scale (FAS). In relation to other anxiety symptoms, including social anxiety, Lebowitz and colleagues also developed two parallel accommodation measures; the Family Accommodation Scale—Anxiety (FASA; Lebowitz et al., 2013) and the Family Accommodation Scale Anxiety—Child Report (FASA-CR; Lebowitz et al., 2015). The two scales share parallel items assessing accommodating behaviors (first nine items) in addition to the distress of accommodators and negative short-term consequences of not accommodating (four items). The FASA-CR adds three supplemental items regarding the child’s beliefs about accommodation. The main differences between the two scales are that the FASA is reported by accommodators, i.e., parents of anxious children, while the FASA-CR is reported by anxious children themselves. Both scales established two related PAR and MOD factor structures, with evidence of good psychometric properties including internal consistency, convergent and divergent validity, and test–retest reliability (Lebowitz et al., 2019). In addition, the factor structures and psychometric properties of the FASA were previously identified on a cross-cultural sample of accommodators from the United States and Israel (Lebowitz et al., 2013), supporting the potential of the cross-cultural properties of FASA and FASA-CR.

Joogoolsingh et al. (2015) developed another measure of SAD accommodation, derived from the FAS, called the Social Anxiety Accommodate Scale (SAAS). The SAAS only includes items specific to social anxiety accommodating behaviors. Considering that adults might not live with their families, accommodators are thus defined as anyone who provides symptom accommodation for the sufferers. To date, there are no reported data on the factor structure or psychometric properties of the SAAS.

The current study aims to modify a well-established measure to assess symptom accommodation in relation to social anxiety symptoms among Chinese adults and test the factor structure and psychometric properties of the modified measure. Specifically, we choose to modify FASA-CR, instead of the SAAS, because of the following characteristics: (1) the target of anxiety disorders makes it easy to adapt to SAD; (2) the reporters of the measure are sufferers, which brings clinical convenience, as adult sufferers are usually the direct source of information regarding their social anxiety symptoms and related accommodation behaviors; and (3) adults may live alone or seek help alone. Finally, the FASA-CR demonstrated a good two-factor structure and sound psychometric properties, in contrast to the SAAS (Lebowitz et al., 2019).

We therefore hypothesized: (1) the two correlated PAR and MOD factor structure will be validated among Chinese adults; (2) the modified measure will demonstrate good internal consistency, measured by Cronbach’s α; and (3) the total score of the modified measure will be significantly associated with SAD symptom severity, SAD-related impairment, and anxiety severity (convergent validity), but not with ratings of depression severity (divergent validity). We did not make specific hypotheses about measurement invariance across gender given a lack of prior evidence.

## Materials and Methods

### Participants and Procedures

Participants were undergraduate students recruited from a large, public university in east China. The study procedure was approved by the Shanghai Normal University Research Ethics Committee. Instructors administered the survey at the end of Psychology or Education courses. Participation was voluntary. Students provided informed written consent and completed a battery of measures by paper-and-pencil. All measures were written in Chinese. The survey took approximately 25 min to complete with all participants receiving a small remuneration. A total of 387 students completed the survey.

Participants were excluded if they (a) did not endorse any social anxiety symptoms and (b) had more than 20% missing values on the Family Accommodation Scale Anxiety—Adult Report (FASA-AR; Lebowitz et al., 2013). The final sample comprised 111 males and 264 females, with a mean age of 19 years (range = 18–26, SD = 1.13). Using a cutoff score of 60 for significant social anxiety (Mennin et al., 2002) on the Liebowitz Social Anxiety Scale (LSAS-SR; Liebowitz, 1987), 81 participants (21.6%) reported elevated social anxiety symptoms.

### Measures

#### Family Accommodation Scale Anxiety—Adult Report

The FASA-AR is a 16-item self-report measure of anxiety symptom accommodation during the past month. It was adapted from and mirrored the FASA-CR (Lebowitz et al., 2015), which is a 16-item self-report questionnaire assessing anxiety symptom accommodation provided by parents. Family Accommodation Scale Anxiety—Child Report consisted of two parts. The first part includes nine items that assesses the frequency of symptom accommodation. Two subscales are generated: PAR and MOD. All nine items in this part are rated on a 0–4 rating scale ranging from *very rarely* to *very often*. The second part includes supplemental questions associated with symptom accommodation. Among them, one item asks about accommodators’ distress (as perceived by the subject) related to accommodation; three items query consequences of not accommodating; and three other items query participants’ beliefs regarding accommodation. All seven items in the second part are rated on a 0–4 rating scale ranging from *strongly disagree* to *strongly agree*. A total accommodation score is calculated by summing the nine items in the first part.

Translation of the original English version of the FASA-CR into Chinese was the first step in creating the FASA-AR. Translation and back translation were carried out by independent bilingual research assistants, in consultation with the developer of the scale, until a satisfactory Chinese version was developed. Next, items were modified to better suit adult responders rather than children, for instance, we replaced the usage of “parent” with “relative or friend” to fit adult lives; the examples helping operationalizing each item fit adult lives. For the current study, FASA-AR items were also adjusted to focus more specifically on social anxiety symptom accommodation, rather than more broadband anxiety symptoms. Finally, accommodators were defined as anyone who provided accommodation for participants’ social anxiety symptoms, including but not limited to (grand)parents, siblings, spouse, friends, and roommates. All modifications were discussed and agreed on by the corresponding author and the last author. Psychometric properties of the FASA-AR are detailed in the text that follows.

#### Liebowitz Social Anxiety Scale

The LSAS-SR (Liebowitz, 1987) is a 24-item self-report measure assessing the severity of social anxiety symptoms. A sample item includes “meeting strangers.” Specifically, the measurement rates the degree of anxiety and related avoidance in 13 social interaction and 11 performance situations. All items are accompanied by two 0–3 rating scales (from *none* to *severe* for assessing degree of anxiety, and from *never* to *usually* for assessing related avoidance). Both anxiety and avoidance rating items are summed for the total score. The LSAS-SR demonstrates good psychometric properties among Chinese SAD patients and university students (Zhang, 2004; Pan et al., 2006). Internal consistency for the current sample was 0.95.

#### Depression Anxiety Stress Scale-21

The Depression Anxiety Stress Scale-21 (DASS-21; Lovibond and Lovibond, 1995) is a 21-item measure of depression, anxiety, and stress. Each item is rated on a 0–3 rating scale ranging from *did not apply to me at all* to *applied to me very much, or most of the time*. Only two subscales, i.e., anxiety and depression subscales, were used in the present study. A sample item of the anxiety subscale includes “I felt scared without any good reason.” A sample item of the depression subscale includes “I couldn’t seem to experience any positive feeling at all.” Previously, the DASS-21 has established good psychometric properties in Chinese patients, college and community samples (Gong et al., 2010; Wen et al., 2012; Zhang et al., 2016). Internal consistency for the present study was 0.90.

#### Sheehan Disability Scale

The Sheehan Disability Scale (SDS; Sheehan, 1983) is a three-item measure of impairment in school/work, social, and family responsibilities. For the present study, instructions were revised to focus on the impairment specific to social anxiety symptoms. A sample item includes “the social anxiety symptoms have disrupted your social life.” The SDS demonstrated good psychometric properties among Chinese adults (Zhu and Zhong, 2010). Each item is rated on a 0–10 rating scale ranging from *not at all* to *extremely* interfering. Internal consistency for the current study was 0.91.

### Data Analysis

Consistent with previous literature, symptom accommodation behaviors related to social anxiety (i.e., the nine items of the first part of the FASA-AR) were of primary interest for the goals of the current study. Confirmatory factor analysis (CFA), with weighted least squares means and variance adjusted estimation (WLSMV), was conducted on the nine symptom accommodation items of the FASA-AR, using M-plus 7.5 (Muthén and Muthén, 2015). Weighted least squares means and variance adjusted estimation was employed because it was more proper for categorical items with five (as for the FASA-AR) or less options than estimation of maximum likelihood (Finney and DiStefano, 2006; Bandalos, 2014). Four fit indices were adopted to assess the goodness-of-fit of the CFA models: model chi-square (χ^2^), comparative fit index (CFA), Tucker Lewis index (TLI), and root mean square error of approximation (RMSEA). Overall fit criteria of these indices are (Hu and Bentler, 1999; Yu, 2002) non-significant χ^2^; CFI and TLI acceptable if >0.90 and good if >0.94; and RMSEA acceptable if <0.10 and good if <0.05.

Although symptom accommodation reports were corroborated, including PAR and MOD factors (as reviewed in the previous paragraph), previous literature suggested that there are two slightly different two-factor models: model 1, supported by Lebowitz et al. (2019), in which items 1–4 are loaded on PAR and items 5–9 are loaded on MOD; model 2, supported by Lebowitz et al. (2013), where items 1–5 are loaded on PAR and items 6–9 are loaded on MOD. The difference between Bayesian information criterion (BIC) values was employed in order to compare these two competing non-nested models. A BIC difference of 10 represents a 150:1 likelihood (*p* < 0.05) that the model with lower BIC value fits better; the difference between 6 to 10 provides strong support; a difference of more than 10 indicates very strong support (Raftery, 1995). Since BIC value is not provided using WLMSV in M-plus, we computed BIC by estimating the model with Maximum Likelihood estimation (Wang et al., 2013).

After the establishment of the best fitting factor structure model, we conducted a multiple indicators multiple causes (MIMIC) model (Jöreskog and Goldberger, 1975) to test measurement invariance across gender. Then, we utilized the Cronbach’s α to assess internal consistency for the FASA-AR as well as its two subscales. Pearson correlations with LSAS-SR, SDS, DASS-21-anxiety, and DASS-21-depression scores were calculated to test the convergent and divergent validity of the FASA-AR total score. Descriptive statistics including means, standard deviations, ranges, and frequencies were reported for all items of the FASA-AR.

## Results

### Confirming the Two-Factor Model of Symptom Accommodation

Model 1, in which items 1–4 are loaded on PAR and items 5–9 are loaded on MOD, showed good fit: χ^2^ = 104.15, *df* = 26, *p* = 0.000; CFI = 0.98; TLI = 0.97; RMSEA = 0.09, 90% CI = [0.07, 0.11], the standard correlation between PAR and MOD is 0.79. The standardized factor loadings are shown in Table 1. While model 2, in which items 1–5 are loaded on PAR and items 6–9 are loaded on MOD, showed acceptable fit: χ^2^ = 178.14, *df* = 26, *p* = 0.000; CFI = 0.96; TLI = 0.95; RMSEA = 0.13, 90% CI = [0.11, 0.14]. The BIC of model 1 was 8557.06, and the BIC of model 2 was 8605.21. The difference between the two BIC values was 48.15, providing “very strong” support for model 1.

**TABLE 1:** Factor loadings of the Family Accommodation Scale Anxiety—Adult Report.

Item	Participation	Modification
1. Providing reassurance	0.64	
2. Proving items	0.71	
3. Participating in behaviors	0.78	
4. Assisting avoidance	0.80	
5. Avoiding things or places		0.82
6. Modifying routine		0.79
7. Doing things instead of you		0.72
8. Modifying work schedule		0.82
9. Modifying leisure activities		0.87

*All factor loadings *ps* < 0.001.*

### Measurement Variance Across Gender

The MIMIC model showed good fit: χ^2^(33) = 133.92, *p* = 0.000; CFI = 0.98; TLI = 0.97; RMSEA = 0.09, 90% CI = [0.08, 0.11]. Gender had a significant effect on PAR (standardized path coefficient = 0.14, *p* = 0.02), but not MOD (standardized path coefficient = 0.01, *p* = 0.83), suggesting that the indicators of FASA-AR were not invariant across gender.

### Internal Consistency

The internal consistencies for the nine symptom accommodation items of the FASA-AR as well as the two subscales (PAR and MOD) were as follows: Cronbach’s α = 0.88, 95% CI [0.86,0.90] for total symptom accommodation; Cronbach’s α = 0.78, 95% CI [0.74,0.81] for the PAR subscale; Cronbach’s α = 0.86, 95% CI [0.83,0.88] for the MOD subscale. Taken together, the internal consistencies for the FASA-AR and its subscales were good.

### Convergent/Divergent Validity

Pearson correlation coefficients used to rate convergent and divergent validity are shown in Table 2. In examining convergent validity, a significant positive relationship was observed between the FASA-AR total score and the LSAS, SDS, and DASS 21-Anxiety scores. When assessing divergent validity, the FASA-AR total score was found to not significantly relate to the DASS 21-Depression score.

**TABLE 2:** Correlation coefficients for study variables.

	FASA-AR_Total	LSAS_Total	SDS_Total	AnxDASS	DepDASS
FASA-AR_Total	–	0.16**	0.19**	0.16**	0.05
LSAS_Total		–	0.54**	0.48**	0.41**
SDS_Total			–	0.41**	0.37**
AnxDASS				–	0.51**
DepDASS					–

FASA-AR_Total, Family Accommodation Scale Anxiety—Adult Report total score; LSAS_Total, Liebowitz Social Anxiety Scale—Self-Report total score; SDS_Total, Sheehan Disability Scale total score; AnxDASS, Depression-anxiety-stress scale_Anxiety subscale score; DepDASS, Depression-anxiety-stress scale_Depression subscale score. **p < 0.01.

### Descriptive Statistics

The means, standard deviations, ranges, and frequencies of each FASA-AR item are presented in Table 3. Nearly all participants (94.7%) endorsed at least some level of accommodating, and most (82.4%) reported both participation in symptom-related behavior and modification of functioning. Among those reporting being accommodated, reassurance (91.0%) occurred with the most frequency. Additionally, more than 70% of participants reported that their accommodators helped participate in behaviors (78.0%), assisted in avoidance (75.5%), and did things for the participants (72.7%). The least frequently endorsed accommodation behavior was changing their work schedule because of the participants’ social anxiety (55.5%).

**TABLE 3:** Individual items endorsed on the Family Accommodation Scale Anxiety—Adult Report.

FASA-AR	*M*	SD	Frequency of Endorsement					
			0	1	2	3	4	Missing values
1	1.86	1.15	52 (13.9%)	94 (25.1%)	112 (29.9%)	89 (23.7%)	28 (7.5%)	0
2	0.89	0.97	164 (43.7%)	121 (32.3%)	64 (17.1%)	21 (5.6%)	5 (1.3%)	0
3	1.40	1.12	98 (26.1%)	107 (28.5%)	103 (27.5%)	53 (14.1%)	13 (3.5%)	1
4	1.26	1.06	107 (28.5%)	123 (32.8%)	89 (23.7%)	48 (12.8%)	6 (1.6%)	2
5	1.03	0.97	141 (37.6%)	109 (29.1%)	98 (26.1%)	24 (6.4%)	2 (0.5%)	1
6	0.95	1.00	161 (42.9%)	107 (28.5%)	72 (19.2%)	33 (8.8%)	1 (0.3%)	1
7	1.25	1.09	117 (31.2%)	106 (28.3%)	101 (26.9%)	41 (10.9%)	9 (2.4%)	1
8	0.81	0.94	178 (47.5%)	119 (31.7%)	51 (13.6%)	26 (6.9%)	1 (0.3%)	0
9	0.94	0.99	162 (43.2%)	102 (27.2%)	87 (23.2%)	19 (5.1%)	5 (1.3%)	0
10	0.85	0.84	147 (39.2%)	152 (40.5%)	59 (15.7%)	16 (4.3%)	0 (0.0%)	1
11	1.12	1.01	121 (32.3%)	134 (35.7%)	77 (20.5%)	40 (10.7%)	3 (0.8%)	0
12	0.83	0.85	156 (41.6%)	141 (37.6%)	63 (16.8%)	15 (4.0%)	0 (0.0%)	0
13	1.22	1.08	116 (30.9%)	120 (32.0%)	81 (21.6%)	52 (13.9%)	5 (1.3%)	1
14	2.54	1.01	21 (5.6%)	39 (10.4%)	71 (18.9%)	201 (53.6%)	4 (10.7%)	3
15	2.37	1.05	27 (7.2%)	43 (11.5%)	104 (27.7%)	163 (43.5%)	37 (9.9%)	1
16	1.70	0.96	37 (9.9%)	125 (33.3%)	138 (36.8%)	65 (17.3%)	10 (2.7%)	0

Only a few participants (4.3%) agreed that accommodators get upset when they offer accommodation. Fewer than a quarter of participants (21.3%) reported negative consequences of not accommodating. Regarding beliefs and attitudes toward symptom accommodation, most participants (64.3%) reported feeling less anxious when they were accommodated, about half of participants (53.4%) agreed that they would feel less anxious in the future if accommodators kept accommodating, and only 20% of the participants endorsed the belief that accommodators should accommodate less when they are anxious.

## Discussion

The current study represents an initial attempt to assess symptom accommodation of Chinese adults with SAD symptoms. Consistent with findings in other cultures (Norman et al., 2015; Lebowitz et al., 2016; Kagan et al., 2017), symptom accommodation is prevalent and occurs for the majority of participants. It is understandable in a culture that highlights relationships between people, symptom accommodation communicates sensitivity to social cues. However, in the case of social anxiety symptom accommodation, this culturally advocated behavior could act as a maintaining factor for the development of SAD and/or serve as an obstacle to the treatment of social anxiety symptoms, substantiating the importance of paying special attention to the phenomenon in the clinical assessment and the utility of having a Chinese measure to assess these detrimental behaviors.

We adapted the FASA-CR into FASA-AR for assessing symptom accommodation among adults. The FASA-CR is the only sufferers-reported and well-established measure assessing symptom accommodation in relation to anxiety disorders, including SAD. Considering the reality that adults usually seek help alone, as well as adult sufferers are likely to have the best perspective on the symptom accommodation, we adapted the FASA-CR for Chinese adult sufferers. As the first Chinese instrument assessing symptom accommodation among adults, the FASA-AR could satisfy the immediate needs. For a more comprehensive understanding of symptom accommodation, measuring the phenomenon from the perspectives of accommodators and clinicians would be an important next step.

Our factor analysis confirmed that the Chinese FASA-AR comprised two correlated factors: participation in symptom-related behaviors and modification of functioning. Previous findings (Lebowitz et al., 2013, 2019) differed on which latent factor item 5 (avoiding doing things, going places or being with people because of the symptoms) loaded. Our results supported the model distributing this item to the modification of functioning factor (Lebowitz et al., 2019). Our MIMIC model indicated that the participation in the symptom-related behaviors subscale did not demonstrate measurement invariance across gender. Therefore, gender differences of this subscale should be interpreted with caution.

Overall, the current results added to the literature that the two-factor structure of symptom accommodation applied to Chinese adults with social anxiety symptoms. It warrants further direct cross-cultural comparison of symptom accommodation in future studies. It is also important to note that the current study is a top-down test, which facilitated capturing the universal aspects of symptom accommodation. Cross-cultural study on the presentation of social anxiety (Zhu et al., 2014) revealed that Chinese adults who were anxious in social situations tended to present higher levels of anxiety about causing discomfort to others, a related but distinct dimension from social interaction anxiety defined by the *Diagnostic and Statistical Manual of Mental Disorders, Fifth Edition* (DSM-5), compared with Euro-Canadian outpatients. Accordingly, accommodators should convey empathy for the anxious individual while encouraging them to gradually face feared situations.

Convergent validity for the FASA-AR was supported by significant positive associations with social anxiety symptom severity, SAD-related functioning impairments, and anxiety. The results, taken together, reconfirmed that symptom accommodation works as more of a hindrance than a help, by preventing socially anxious individuals from being exposed to corrective learning experiences (La Buissonnière-Ariza et al., 2017), albeit the intent is good. Our cross-sectional design can only reveal the bidirectional associations between social anxiety symptom severity and symptom accommodation, and SAD-related impairment and symptom accommodation. Future studies should further investigate the role symptom accommodation might play on the clinical course of SAD with a longitudinal design. The FASA-AR was correlated with anxiety symptoms, which is not surprising because social anxiety is one domain of the anxiety disorder spectrum. Furthermore, anxiety broadly might be a driving force of symptom accommodation. When exposed to or anticipating feared social situations, the heightened anxiety may make accommodators perceive the need to provide accommodation to alleviate the distress of the individuals with social anxiety symptoms (Wu et al., 2015).

Cross-cultural similarities were found on the most and least frequent accommodating behaviors, distress of accommodators perceived by sufferers, consequences of not providing accommodation, and sufferers’ belief about accommodation between our sample and United States samples in previous studies (Joogoolsingh et al., 2015; Lebowitz et al., 2015). It is notable that these similarities occurred on the report by sufferers. Lebowitz et al. (2015) found that United States accommodators tended to report higher level of accommodation than sufferers being accommodated. Future studies should compare the data from Chinese accommodators and sufferers.

Our preliminary findings showed promise for the necessity and validity of the FASA-AR among Chinese adults; however, several limitations should be noted. First, our sample only included college students, limiting generalizability. Future studies should test the scale in community participants or clinical samples. Second, we did not examine test-retest reliability. Lebowitz et al. (2019) examined the test–retest reliability of FASA-CR, from which our FASA-AR was adapted, among anxious youth and found that reliability was good for adolescents (>12 years), suggesting the test–retest reliability of FASA-AR among adults might also be in the acceptable range. Third, our cross-sectional design did not allow us to test the sensitivity of the FASA-AR to treatment. Fourth, we solely relied on self-report measures to assess symptom accommodation; more objective measures and reports provided by those doing the accommodation are recommended in further studies. Finally, we could not guarantee measurement invariance across gender or other potential variables of interest. Thus, caution should be given when interpreting results.

Ultimately, symptom accommodation deserves careful attention in the clinical assessment and treatment. It is culturally valued but problematic. Recognizing the prevalence of accommodation and identifying concrete accommodating behavior types would help Chinese clinicians target the behaviors effectively during treatment of social anxiety symptoms. It has been found that reducing symptom accommodation has effectively led to symptom improvement among anxious individuals, including those with SAD (Lebowitz et al., 2014, 2020). For those with social anxiety symptoms, whose natural eagerness for assistance, efforts trying to reduce symptom accommodation during treatment would be expected to be opposed, as well as for relatives or other accommodators, who are an important part of the treatment, careful explanations (reducing accommodation is helpful and not against cultural norms) are in great need in the psychoeducation. Efforts should be given to help accommodators coordinate the accommodation reduction with the cultural norm of helping sufferers. It is notable that completing the FASA-AR itself could serve as the first step to obtain therapeutic gains, because it could bring the awareness of the maladaptive behaviors and establish the foundation for the gradual removal of accommodation (Wu et al., 2015). Overall, the FASA-AR demonstrated a clear two-factor structure, and possessed good psychometric properties, reflecting its utility for measuring symptom accommodation.

## Data Availability Statement

The datasets generated for this study are available on request to the corresponding author.

## Ethics Statement

The studies involving human participants were reviewed and approved by the institutional review board at Shanghai Normal University. The patients/participants provided their written informed consent to participate in this study.

## Author Contributions

CL drafted the manuscript. XZ designed the study, collected the data, did data analysis, and revised the manuscript. EL and LW revised the manuscript. ES contributed to the conception and design of the study, as well as revised the manuscript.

## Conflict of Interest

ES receives book royalties from Springer, Elsevier, Lawrence Erlbaum, Wiley, American Psychological Association, Oxford, and Jessica Kingsley. He is a consultant for Levo Therapeutics. He receives grant funding from NIH, Red Cross, ReBuild Texas, Texas Higher Education Coordinating Board, and Greater Houston Community Foundation. He receives personal fees for doing trainings on psychotherapy for OCD with the International OCD Foundation. EL receives royalties from Oxford and Wiley, and grant funding from NIMH. The remaining authors declare that the research was conducted in the absence of any commercial or financial relationships that could be construed as a potential conflict of interest.
